# Interpretable analysis of smartphone addiction status and its associated factors among college students

**DOI:** 10.3389/fpsyt.2026.1850706

**Published:** 2026-06-24

**Authors:** Yuanning Li, Najie Zhao, Yanyan Wang

**Affiliations:** 1 School of Physical Education, Yanshan University, Qinhuangdao, Hebei, China; 2 School of Medicine, Weifang University of Science and Technology, Weifang, Shandong, China; 3 Department of Emergency Medicine, Zhucheng People’s Hospital (Affiliated with Shandong Second Medical University), Weifang, Shandong, China

**Keywords:** college students, machine learning, predictive modeling, smartphone addiction, XGBoost

## Abstract

**Background:**

This study aimed to develop and validate a risk prediction model for smartphone addiction among college students using an extreme gradient boosting (XGBoost) algorithm, and to identify key factors associated with this behavioral pattern.

**Methods:**

A cross-sectional survey was conducted among 2,761 college students. The XGBoost machine learning algorithm was applied to analyze the dataset, enabling the identification of variables associated with smartphone addiction and the ranking of their relative contributions based on feature importance scores.

**Results:**

The prevalence of smartphone addiction in this sample was approximately 22.24%. According to the XGBoost model, predictors ranked by descending feature importance scores were as follows: Loneliness (0.437), Monthly household income (0.067), Age (0.056), Place of residence (0.056).

**Conclusions:**

Smartphone addiction among college students represents a notable public health concern requiring sustained attention. University educators and administrators are encouraged to prioritize factors showing robust statistical associations with smartphone addiction—including loneliness, monthly household income, age and place of residence—implement routine screening for problematic smartphone use, and develop personalized intervention strategies aligned with individual risk profiles.

## Introduction

Smartphone addiction, also referred to as smartphone dependence, problematic smartphone use, excessive smartphone use, or nomophobia, is a behavioral disorder characterized by compulsive smartphone engagement that significantly impairs daily functioning, interpersonal relationships, occupational performance, and overall well-being (1, 2). Although not currently classified under the “Substance-Related and Addictive Disorders” category in the *Diagnostic and Statistical Manual of Mental Disorders, Fifth Edition* (DSM-5), smartphone addiction is widely recognized as a form of behavioral addiction (3). It exhibits core diagnostic features consistent with established addiction models, including salience, tolerance, withdrawal symptoms, interpersonal conflict, and relapse (4, 5). Notably, accumulating evidence indicates substantial overlap between behavioral and substance addictions across clinical presentations, cognitive processes, neurobiological mechanisms, and neuroimaging profiles (6).

Given its high prevalence, rapid escalation, and substantial adverse consequences, smartphone addiction has emerged as a global public health concern (7). This issue is particularly pronounced among university students in China. A national survey reported that approximately 25% of Chinese college students exhibit problematic smartphone use (8). Compared with other populations, university students typically possess greater discretionary time (9), yet often demonstrate psychological immaturity and limited self-regulatory capacity—factors that collectively heighten vulnerability to smartphone addiction. Prolonged excessive smartphone use has been associated with detrimental effects on both physical and mental health, including poor sleep quality and elevated depressive symptoms (10, 11). In light of the widespread smartphone engagement and its documented harms in this population, elucidating the underlying triggers and mechanistic pathways is critically important for developing targeted prevention and intervention strategies. Although existing studies have investigated the factors influencing smartphone addiction among college students, the majority primarily rely on conventional regression models for analysis (12–15). These methods typically operate under linear or generalized linear assumptions, making it difficult to effectively capture complex non-linear relationships and higher-order interactions among variables. Furthermore, they are sensitive to multicollinearity, resulting in limited predictive accuracy and model generalizability. XGBoost is an advanced ensemble machine learning algorithm that constructs base learners by optimizing a regularized objective function. To enhance computational efficiency and generalization performance while effectively mitigating overfitting, the algorithm incorporates techniques such as pre-sorting and weighted quantile sketching (16). SHAP (Shapley Additive exPlanations) values, grounded in cooperative game theory, quantify the magnitude and direction of each feature’s contribution to model predictions, thereby providing an intuitive and interpretable assessment of feature importance (17). Within this methodological framework, the present study employs an XGBoost model integrated with SHAP-based interpretation to systematically investigate the determinants and interaction patterns underlying smartphone addiction among college students. This approach aims to deliver actionable insights for educators and student support professionals, facilitate the early identification of high-risk individuals, and inform the development of targeted prevention and intervention strategies to mitigate the prevalence and adverse impacts of smartphone addiction in this population.

## Methods

Between March and April 2026, college students were recruited from five universities across Shandong, Hunan, and Hebei provinces using a convenience sampling approach. Data were collected via anonymous self-administered questionnaires distributed by class advisors. Recruitment and data collection continued until no new submissions were received for seven consecutive days.

Inclusion criteria: (1) full-time enrollment at a participating university; (2) sufficient proficiency in Mandarin Chinese to independently comprehend and complete the survey; and (3) willingness to provide informed consent.

Exclusion criteria: (1) a documented history of psychiatric disorders; and (2) being on medical leave or academic suspension during the data collection period.

### General information and questionnaire data

#### Demographic characteristics

Baseline sociodemographic characteristics were recorded across 11 items, including gender, age, et al. educational level, mother’s education level and father’s education level et al.

#### Smartphone Addiction Scale-Short Version

Smartphone addiction was assessed using the SAS-SV, originally developed by Kwon (18) et al. and subsequently translated and culturally adapted into Chinese by Xiang (19) et al. The scale comprises 10 items rated on a 6-point Likert scale, ranging from 1 (*strongly disagree*) to 6 (*strongly agree*). Total scores range from 10 to 60, with higher scores indicating more severe smartphone addiction. In the present study, a total score >33 was used as the cutoff to identify smartphone addiction. The SAS-SV demonstrated excellent internal consistency reliability in this sample, with a Cronbach’s α coefficient of 0.878.

#### The University of California at Los Angeles Loneliness Scale (UCLA-20)

UCLA-20, developed by Russell et al. (20), was used to assess loneliness. The scale comprises 20 items, 9 of which are reverse-scored. Responses are rated on a 4-point Likert scale ranging from 1 (“never”) to 4 (“always”), yielding a total score ranging from 20 to 80. Higher scores indicate greater levels of loneliness. Consistent with previous studies (21), a cutoff score of ≥44 was adopted to classify participants as experiencing loneliness. In the present study, the internal consistency of the UCLA-20 was excellent, with a Cronbach’s α coefficient of 0.870.

### Data collection

Data collection was conducted using an electronic questionnaire. The survey link or QR code was distributed to college students via class counselors. Following a clear explanation of the study objectives, anonymity and confidentiality measures, and the voluntary nature of participation, participants provided informed consent and completed the survey independently. All responses were automatically submitted in real time to the online database. Should any participant encounter difficulties with question comprehension or platform navigation, research staff provided strictly neutral, non-directive technical assistance to ensure data accuracy without introducing response bias.

### Sample size

The sample size was determined based on the events-per-variable (EPV) principle, which recommends a minimum of 10 outcome events per predictor variable to ensure model stability (22). Accounting for an anticipated attrition rate of 10%–20% and 11 candidate variables planned for multivariable modeling, the minimum required sample size was calculated to be 122 participants. A total of 2,785 questionnaires were distributed, and 2,761 valid responses were obtained, yielding a valid response rate of 99.14%.

### Statistical analysis

This study was conducted in a Python 3.9 environment, utilizing the Pandas, NumPy, and XGBoost libraries for predictive modeling and statistical computation. After rigorous data cleaning, the analytical dataset comprised 2,761 complete observations across 11 predictor variables (including both continuous and categorical features) with no missing values.To reduce model complexity, enhance interpretability, and mitigate overfitting risks, we first employed LASSO (Least Absolute Shrinkage and Selection Operator) regression for key variable selection, leveraging its L1-regularization property to shrink irrelevant coefficients toward zero. The dataset was randomly split into a training set (80%) and an independent test set (20%). All modeling procedures—including feature selection, hyperparameter optimization, and model training—were performed exclusively within the training set using 5-fold cross-validation to ensure robustness; the test set was held out completely and used solely for unbiased evaluation of the final model performance. Based on the selected features, we systematically developed and compared seven machine learning models: Logistic Regression, Elastic Network, K-Nearest Neighbors (KNN), Decision Tree, XGBoost, Support Vector Machine (SVM), and Random Forest (see **Supplementary Materials**). The XGBoost model, which demonstrated the best overall performance in terms of accuracy, AUC, and F1-score, was ultimately selected to predict and stratify smartphone addiction severity. XGBoost (Extreme Gradient Boosting) minimizes prediction errors by iteratively constructing decision trees in a stage-wise additive manner, where each new tree corrects the residual errors of its predecessors. Its key hyperparameters—including maximum tree depth (max_depth), number of base learners (n_estimators, representing the total number of sequential decision trees), and learning rate (learning_rate, controlling the contribution weight of each tree)—were comprehensively optimized via grid search combined with 5-fold cross-validation within the training set to ensure robust generalization. To strictly prevent information leakage—a critical concern in machine learning pipelines—the feature selection process was fully nested within the cross-validation loop: in each fold, the LASSO model was independently fitted and variables were selected using only the current training subset without any exposure to validation or test data; the selected variables were then consistently applied to both the training and validation subsets of that fold, while the test fold data were completely excluded from all preceding modeling steps, including feature screening and hyperparameter tuning. Following systematic optimization, max_depth = 2 and n_estimators = 26 (i.e., 26 base learners) were identified as the optimal parameters to balance model performance, computational efficiency, and interpretability. The final model was selected based on the highest mean accuracy across all cross-validation folds, with additional consideration of metric stability (low standard deviation across folds).To enhance model interpretability and bridge the gap between black-box predictions and clinical decision-making, SHAP (Shapley Additive exPlanations) analysis, grounded in cooperative game theory, was employed to quantify the magnitude and direction of each feature’s contribution to individual predictions. The cross-validation-guided feature selection process effectively stabilized feature importance rankings across resampling iterations, ensuring the selection of an optimal and reproducible feature subset, thereby simultaneously improving predictive accuracy, model transparency, and clinical interpretability for practical implementation.

## Results

### Demographic characteristics of participants

A total of 2,761 college students were enrolled in this study, comprising 863 males and 1,898 females. Among them, 614 participants (22.24%) were identified as having smartphone addiction. Detailed demographic characteristics are presented in **Table 1**.

**Table 1:** Demographic characteristics of participants.

Variable	Total	No	Yes
Gender			
Male	863	660	203
Female	1898	1487	411
Age(Years)			
<19	1481	1139	342
20-22	1204	944	260
>22	76	64	12
Educational Level			
Associate Degree Student	1291	969	322
Undergraduate Student	1470	1178	292
Mother’s Education Level			
Junior High School or Below	1625	1259	366
Senior High School or Vocational Secondary	849	661	188
College or Above	287	227	60
Father’s Education Level			
Junior High School or Below	1371	1064	307
Senior High School or Vocational Secondary	1028	804	224
College or Above	362	279	83
Place of residence			
Rural	1441	1127	314
Urban	1320	1020	300
Monthly Income(¥)			
<3000	746	561	185
3001-6000	1239	985	254
6001-9000	488	386	102
≥9001	288	215	73
Live on campus			
No	50	34	16
Yes	2711	2113	598
Smoking			
No	2616	2052	564
Yes	145	95	50
Monthly living expenses(¥)			
<1000	767	597	170
1001-2000	1895	1471	424
>2000	99	79	20
Loneliness			
No	1715	1505	210
Yes	1046	642	404

### Risk factors for smartphone addiction among college students

**Figure 1:**
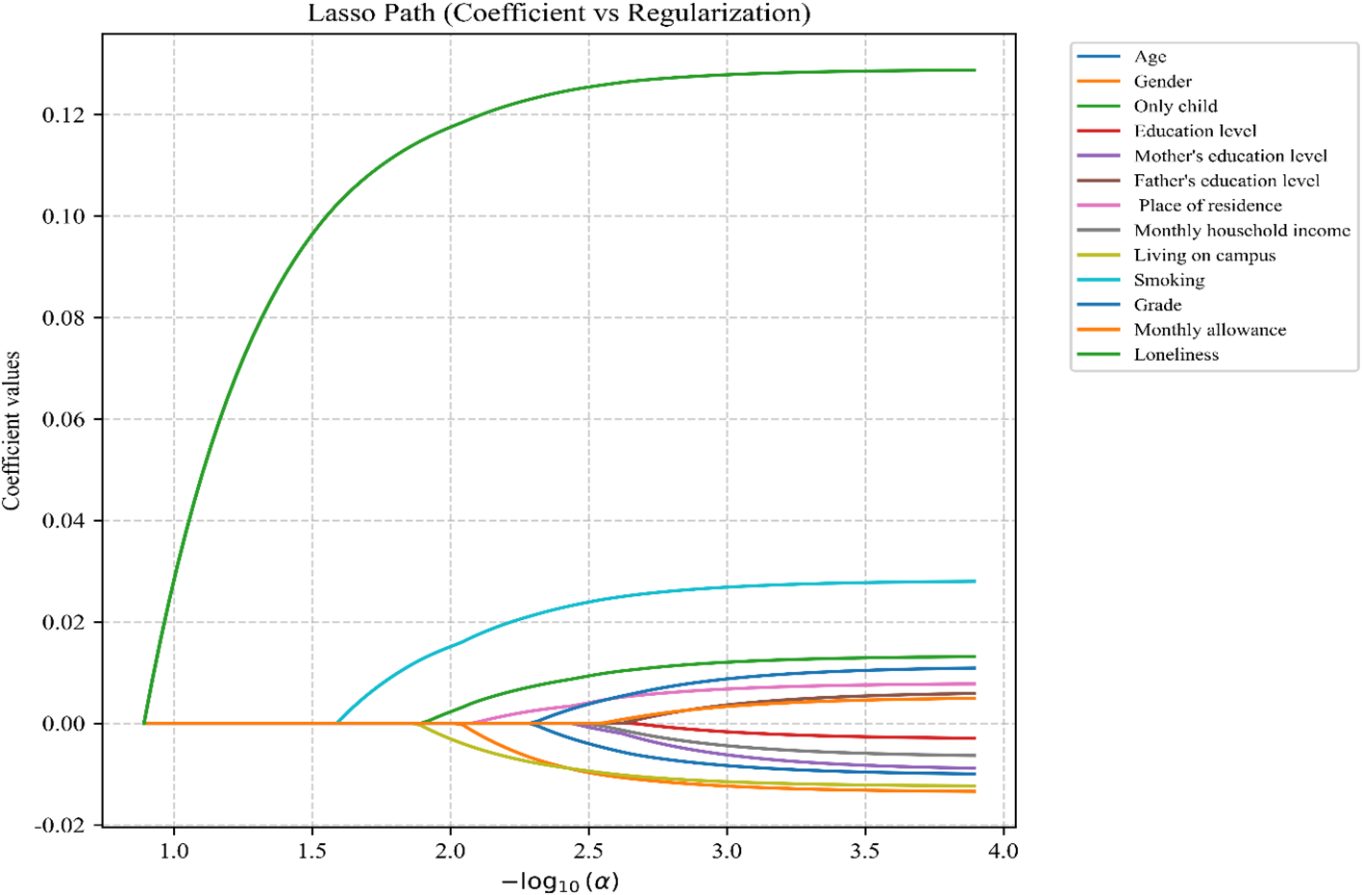
Lasso regression coefficient paths for predictors of smartphone addiction.

**Figure 2:**
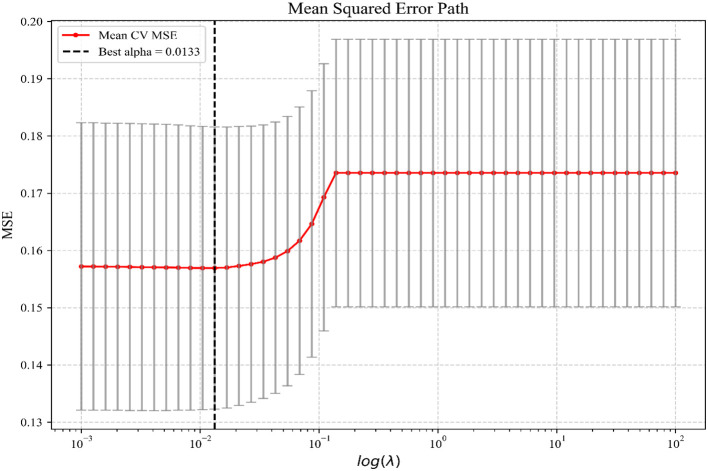
Mean Squared Error (MSE) path with cross-validation for selecting the optimal regularization parameter.

The LASSO regression analysis is illustrated in **Figures 1** and **2**. The optimal regularization parameter (λ) was determined to be 0.013 through cross-validation. Based on this parameter, six predictor variables with non-zero regression coefficients were selected for the final model: Gender, Age, Place of residence, Monthly household income, Smoking, and Loneliness (**Table 2**).

**Table 2:** Results of LASSO regression for predictors associated with smartphone addiction in college students.

Variables	Coefficient	Lambda. min
Gender	-0.013	0.013
Age	-0.006	
Place of residence	0.008	
Monthly Household Income	-0.005	
Smoking	0.077	
Loneliness	0.256	

### XGBoost model results

#### Optimal feature subset

**Figure 3:**
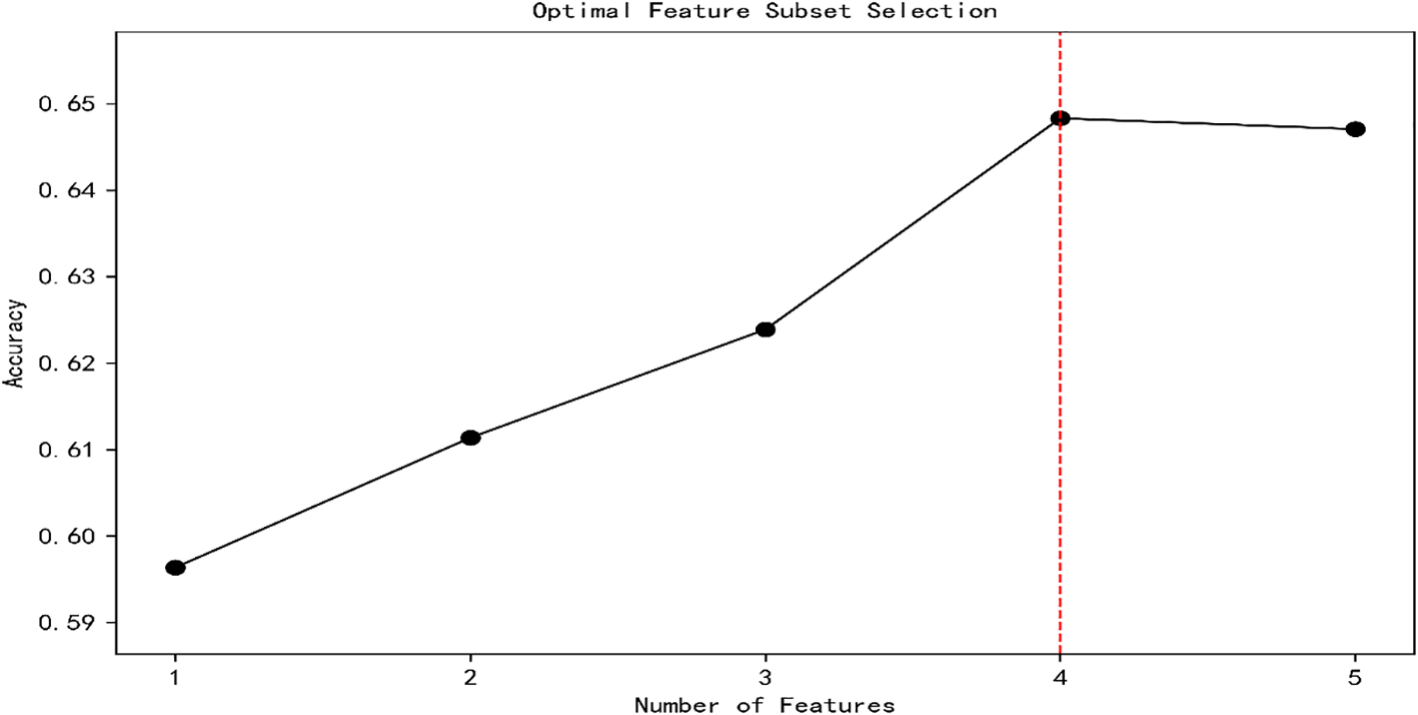
Line graph of the optimal feature subset screening.

Features selected by LASSO regression (non-zero coefficients) served as input variables for the XGBoost classifier. We employed 5-fold cross-validation to systematically evaluate model performance across different feature combinations. Classification accuracy (range: 0–1) was used as the evaluation metric, with a 4-feature subset achieving optimal performance (Accuracy=0.706, Precision=0.638, Specificity=0.873, F1-Score=0.494, 95%CI=0.604-0.643), see **Figure 3**.

#### Feature importance ranking

**Figure 4:**
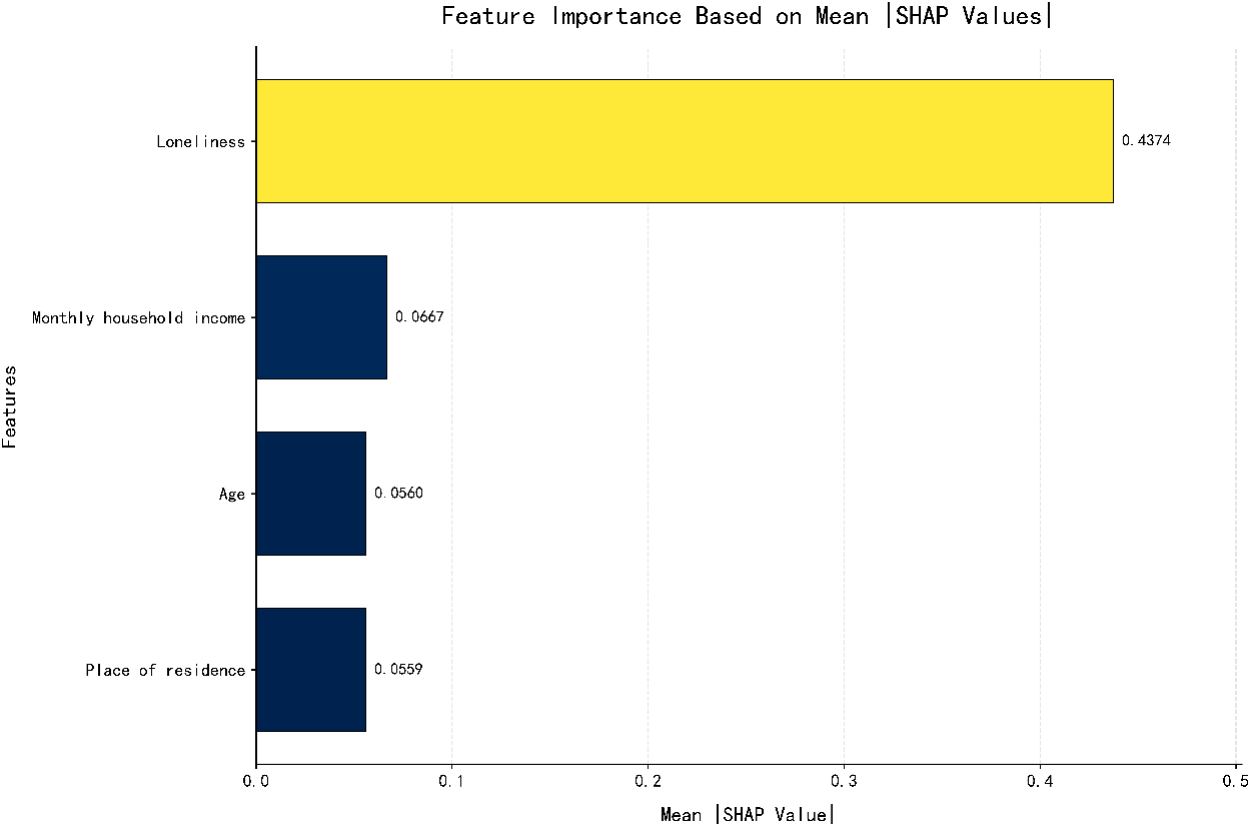
Feature importance ranking.

**Figure 5:**
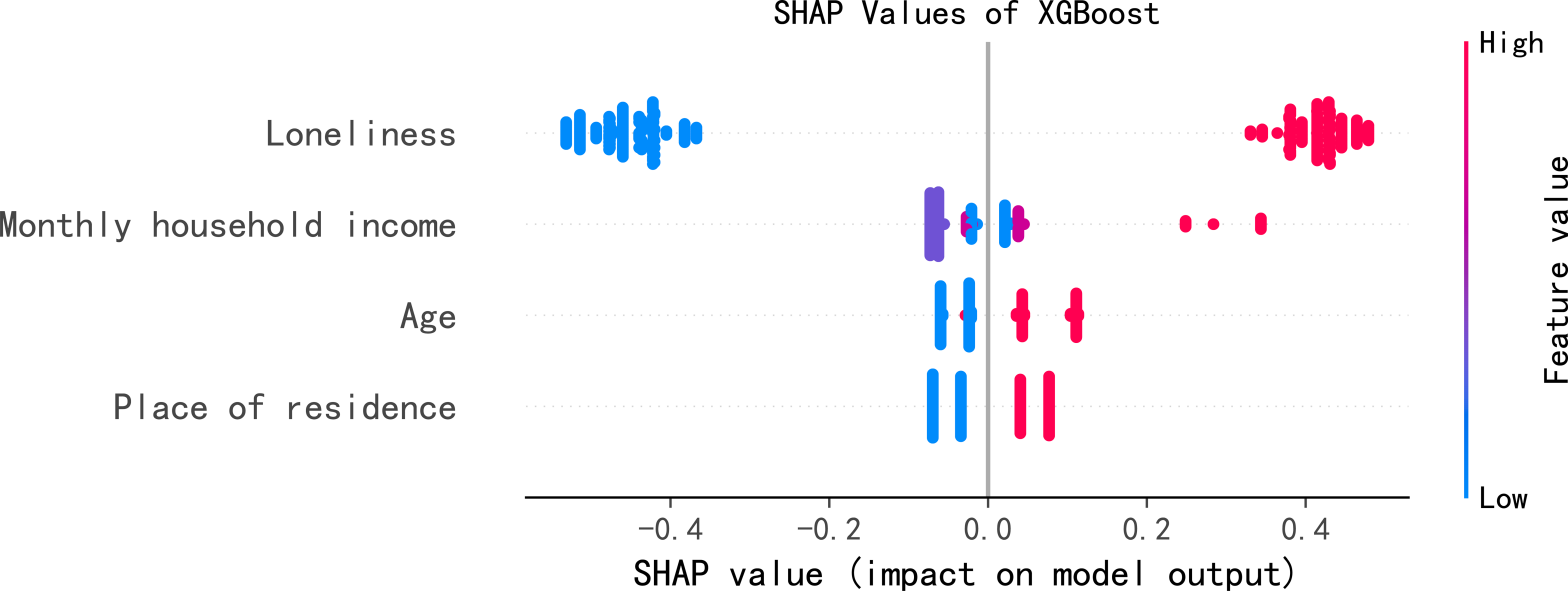
SHAP value (impact on model output). X-axis (SHAP value): Positive values (right side) → Increased predicted risk of mobile phone addiction. Negative values (left side). Decreased predicted risk of mobile phone addiction.Color bar:Blue = Low feature value (lower encoded value). Red = High feature value (higher encoded value).

Categorical variables were encoded as follows: Loneliness (0 = No, 1 = Yes); Monthly household income (0 = ≤3000, 1 = 3001–6000, 2 = 6001–9000, 3 = ≥9000); Age (0 = <19 years, 1 = 20–22 years, 2 = ≥22 years); and Place of residence (0 = Rural, 1 = Urban). The factors influencing smartphone addiction among college students, ranked by mean absolute SHAP values, are: Loneliness (0.437), Monthly household income (0.067), Age (0.056), Place of residence (0.056). The feature levels Loneliness (Yes), monthly household income (≥9000 RMB), age (≥22 years), and place of residence (Urban) all demonstrated strong positive SHAP values, suggesting that these factors substantially elevate the predicted risk of mobile phone addiction in the college student population (**Figures 4**, **5**).

## Discussion

The prevalence of smartphone addiction among college students in this study was 22.24%, slightly lower than the 28.49% reported by Wang et al (23). This discrepancy may be attributable to differences in assessment instruments and regional characteristics. Nevertheless, the observed prevalence remains notably high. Existing literature indicates that smartphone addiction is associated with compromised sleep quality, including insomnia and disrupted sleep patterns. Additionally, prolonged screen time has been linked to elevated psychological stress and co-occurring negative affective states, such as anxiety and depressive symptoms, underscoring potential implications for mental well-being (23). To elucidate variables associated with smartphone addiction and inform targeted intervention strategies, we applied the XGBoost algorithm to identify and rank predictive features. Feature importance analysis revealed the following predictors, ordered by their relative contribution to the model: loneliness, monthly household income, age and place of residence.

Our findings indicate that loneliness is significantly associated with smartphone addiction among college students, a result consistent with previous cross-sectional studies (24, 25). Loneliness is conceptualized as a multifaceted emotional state that emerges when an individual’s needs for intimacy and social connection are inadequately met. According to the Evolutionary Theory of Loneliness (ETL) (26), humans have evolved an inherent drive to maintain social bonds. Perceived social disconnection triggers an aversive emotional experience, which theoretically functions as an adaptive signal motivating individuals to restore and sustain interpersonal ties. As a highly accessible communication medium, smartphones may offer a readily available channel for social engagement. Consequently, college students experiencing loneliness may turn to their devices to facilitate interpersonal connection. This pattern aligns with Media System Dependency Theory (27), which posits that individuals develop reliance on specific media channels when they perceive them as essential or optimal for achieving personal goals, such as social integration. Given the immediacy and convenience of smartphones in fulfilling social needs compared to alternative media, individuals reporting higher levels of loneliness may exhibit a greater tendency toward problematic smartphone use.

Additionally, our findings indicate that monthly household income is significantly associated with smartphone addiction among college students, a result consistent with prior research (28). Students from higher socioeconomic backgrounds typically gain earlier exposure to smart devices and have greater access to feature-rich, high-end smartphones (29). Against the backdrop of rapid advancements in mobile communication technology, high-end devices offer increasingly diverse functionalities and interactive capabilities, which may facilitate prolonged engagement and higher usage frequency. Theoretical perspectives on media and behavioral engagement suggest that enhanced device functionality and accessibility are linked to patterns of intensive use. Our findings further indicate that age is a significant predictor of smartphone addiction among college students. This association may be partially attributable to developmental differences in self-regulation and academic habits. Moreover, the widespread integration of online learning has introduced environmental vulnerabilities; the reduced external supervision inherent in digital classrooms may impair students’ ability to self-monitor device usage (30). College students from urban areas typically experience earlier smartphone adoption and higher device accessibility, which may foster greater daily reliance and more frequent usage patterns. Moreover, as smartphones have become a primary medium for peer socialization, their constant provision of novel information and seamless global connectivity may collectively contribute to an elevated risk of smartphone addiction among urban undergraduates (30).

## Strengths and limitations

This study employed an XGBoost-based approach to predict the risk of smartphone addiction among college students using data from a multi-center survey. Nevertheless, several limitations warrant acknowledgment. First, the reliance on convenience sampling and self-reported measures may introduce selection and recall biases. Second, the cross-sectional design precludes causal inferences regarding the relationships among the examined variables. Third, the predictive model was constrained by a limited set of covariates, and potential class imbalance within the dataset may have affected performance metrics. Additionally, although cross-validation was implemented, the absence of an independent external validation cohort limits the generalizability of the findings. There is also a possibility of information leakage during the feature selection process (e.g., if screening was conducted prior to data splitting), which may have contributed to an overestimation of model performance. Finally, the study did not incorporate biological markers (e.g., genomic data) or certain behavioral covariates (e.g., physical activity), thereby limiting insights into underlying physiological mechanisms and potential confounding pathways. Future research should prioritize longitudinal designs, recruit larger and more diverse cohorts for external validation, and integrate multi-dimensional data (including behavioral and biological indicators) to elucidate causal mechanisms and enhance predictive robustness.

## Conclusion

In this study, an XGBoost model was employed to identify factors associated with smartphone addiction among 2,761 college students. The key predictors identified included loneliness, monthly household income, age and place of residence. Our findings underscore the importance of early psychological screening and assessment for college students by educators and university administrators. Furthermore, targeted supportive interventions should be implemented to address modifiable risk factors, thereby mitigating the risk of smartphone addiction within this population.

## Data Availability

The original contributions presented in the study are included in the article/**Supplementary Material**. Further inquiries can be directed to the corresponding author.
