# Supplementary material for: Interpretable analysis of smartphone addiction status and its associated factors among college students
Source: Front Psychiatry. 2026 Jun 24;17:1850706. doi: 10.3389/fpsyt.2026.1850706 (PMC13341939; doi:10.3389/fpsyt.2026.1850706)
Supplement: Supplementary file 1 [file Table1.docx]

**Table 1. Performance Comparison of Different Machine Learning Models for Smartphone Addiction Prediction**

| Metrics | Logistic Regression | Elastic Network | KNN | Decision Tree | XGBoost | SVM | Random Forest |
| --- | --- | --- | --- | --- | --- | --- | --- |
| Accuracy | 0.690 | 0.640 | 0.609 | 0.7 | 0.706 | 0.640 | 0.671 |
| Precision | 0.590 | 0.496 | 0.422 | 0.621 | 0.638 | 0.493 | 0.550 |
| Recall | 0.429 | 0.692 | 0.263 | 0.403 | 0.403 | 0.324 | 0.429 |
| F1-Score | 0.497 | 0.578 | 0.324 | 0.489 | 0.494 | 0.391 | 0.482 |
| Sensitivity | 0.429 | 0.692 | 0.263 | 0.403 | 0.403 | 0.324 | 0.429 |
| Specificity | 0.834 | 0.611 | 0.800 | 0.864 | 0.873 | 0.815 | 0.805 |
| Kapp | 0.281 | 0.279 | 0.070 | 0.290 | 0.302 | 0.151 | 0.247 |
| 95%CI | (0.614,  0.644) | (0.596,  0.640) | (0.557, 0.664) | (0.613, 0.646) | (0.604,  0.643) | (0.587, 0.636) | (0.600,  0.671) |


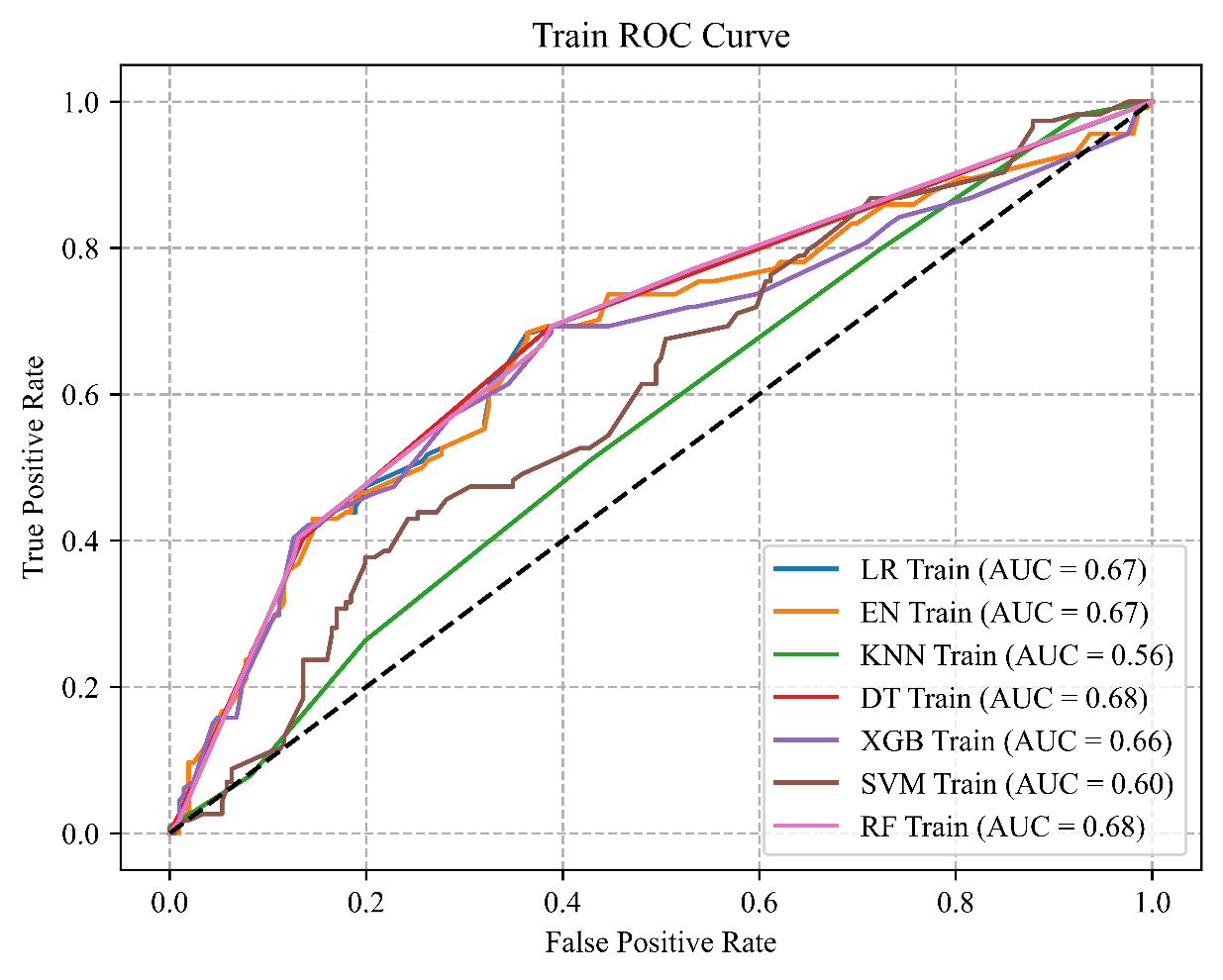


**Figure 1. ROC Performance Evaluation of Different Models on the Train Set**


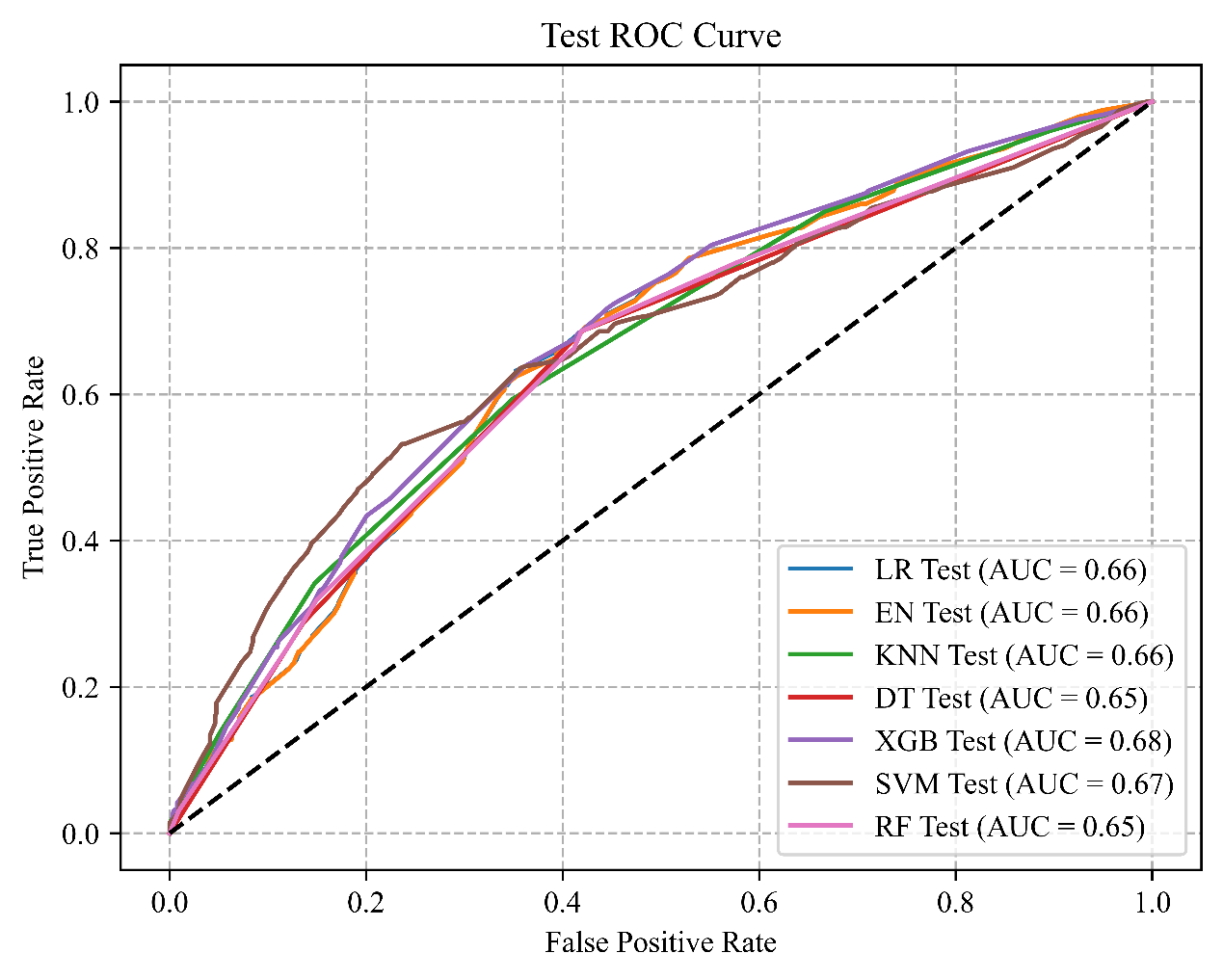


**Figure 2. ROC Performance Evaluation of Different Models on the Test Set**


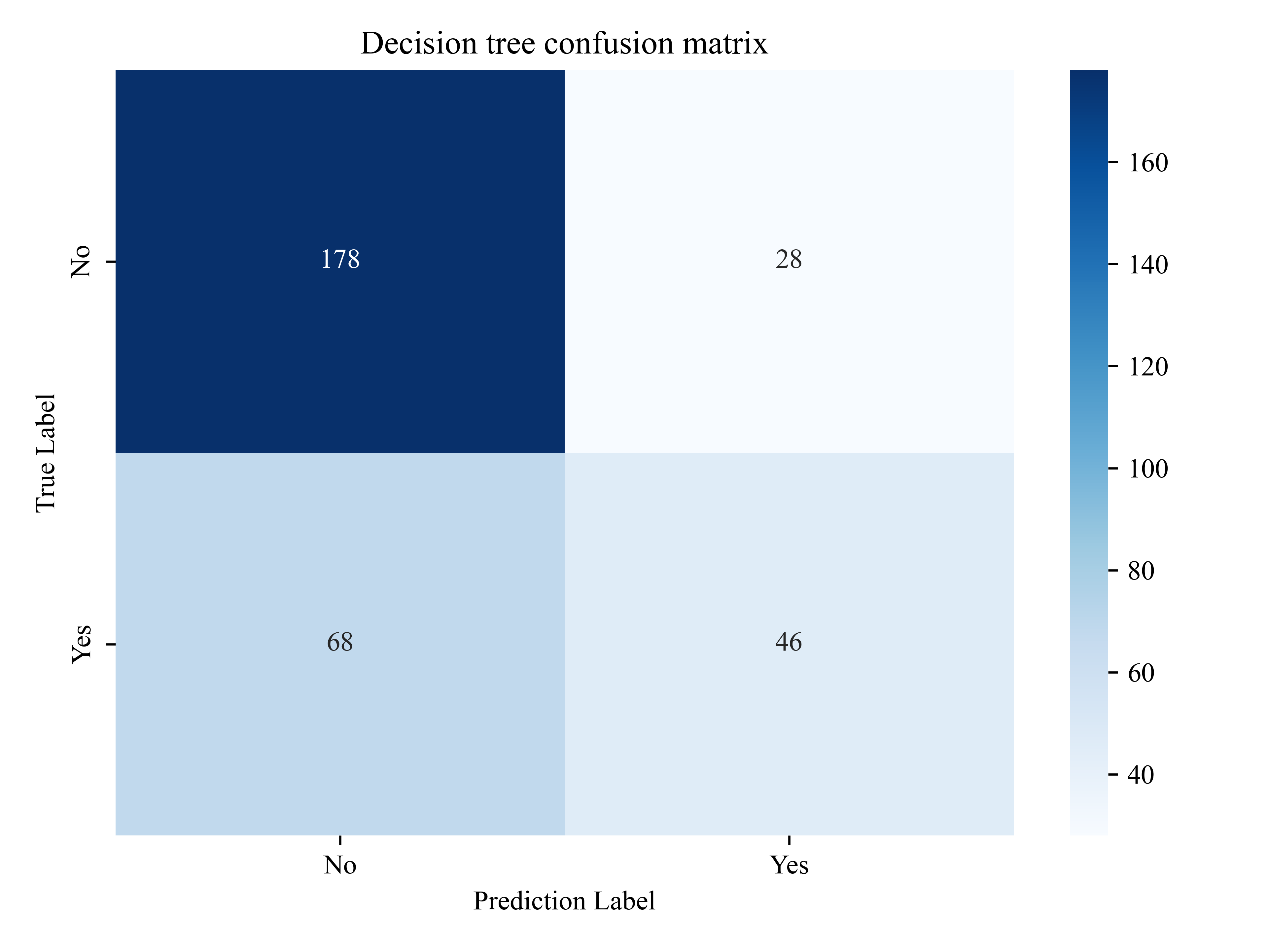


**Figure 3. Decision tree confusion matrix**


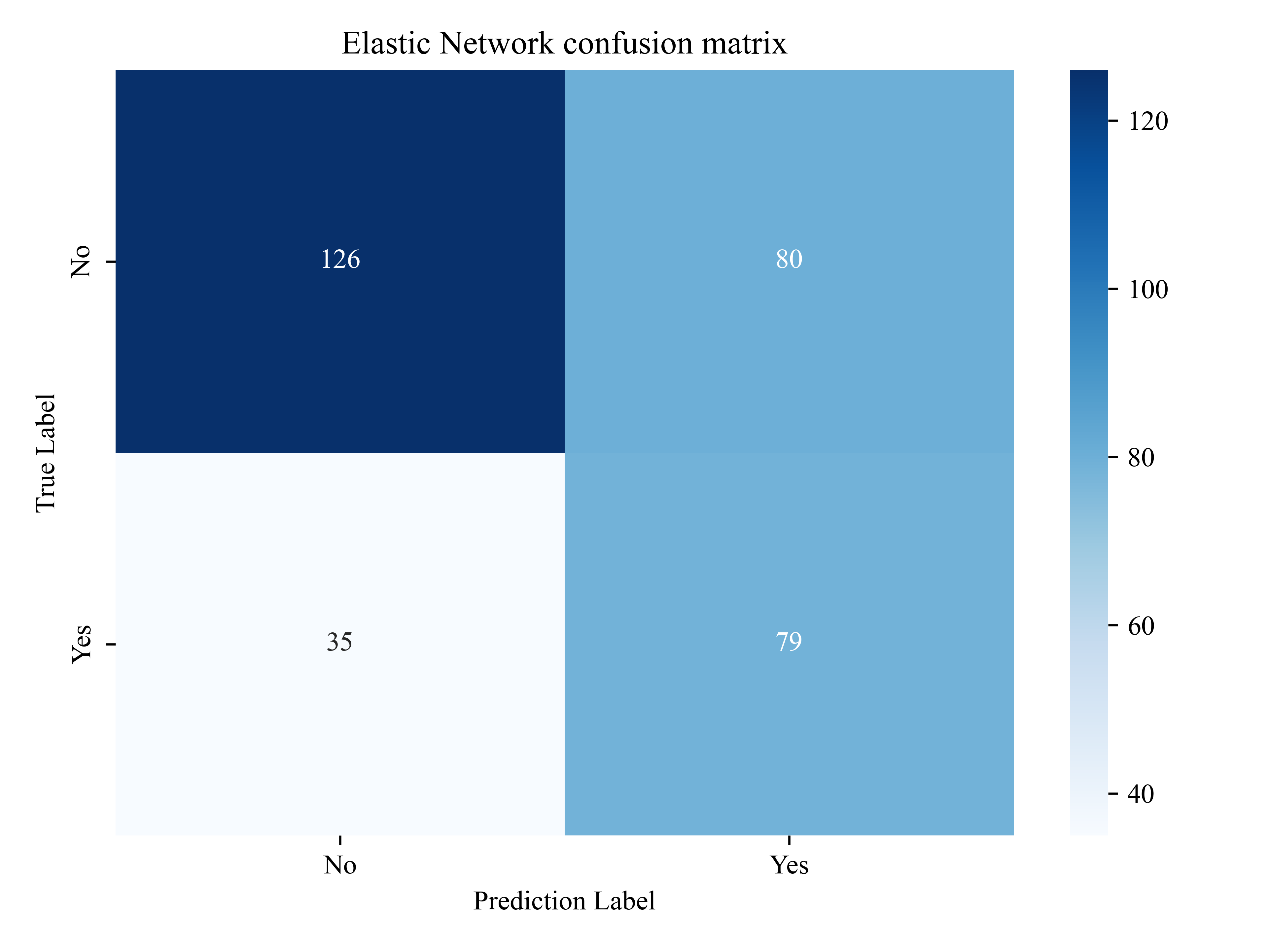


**Figure 4. Elastic Network confusion matrix**


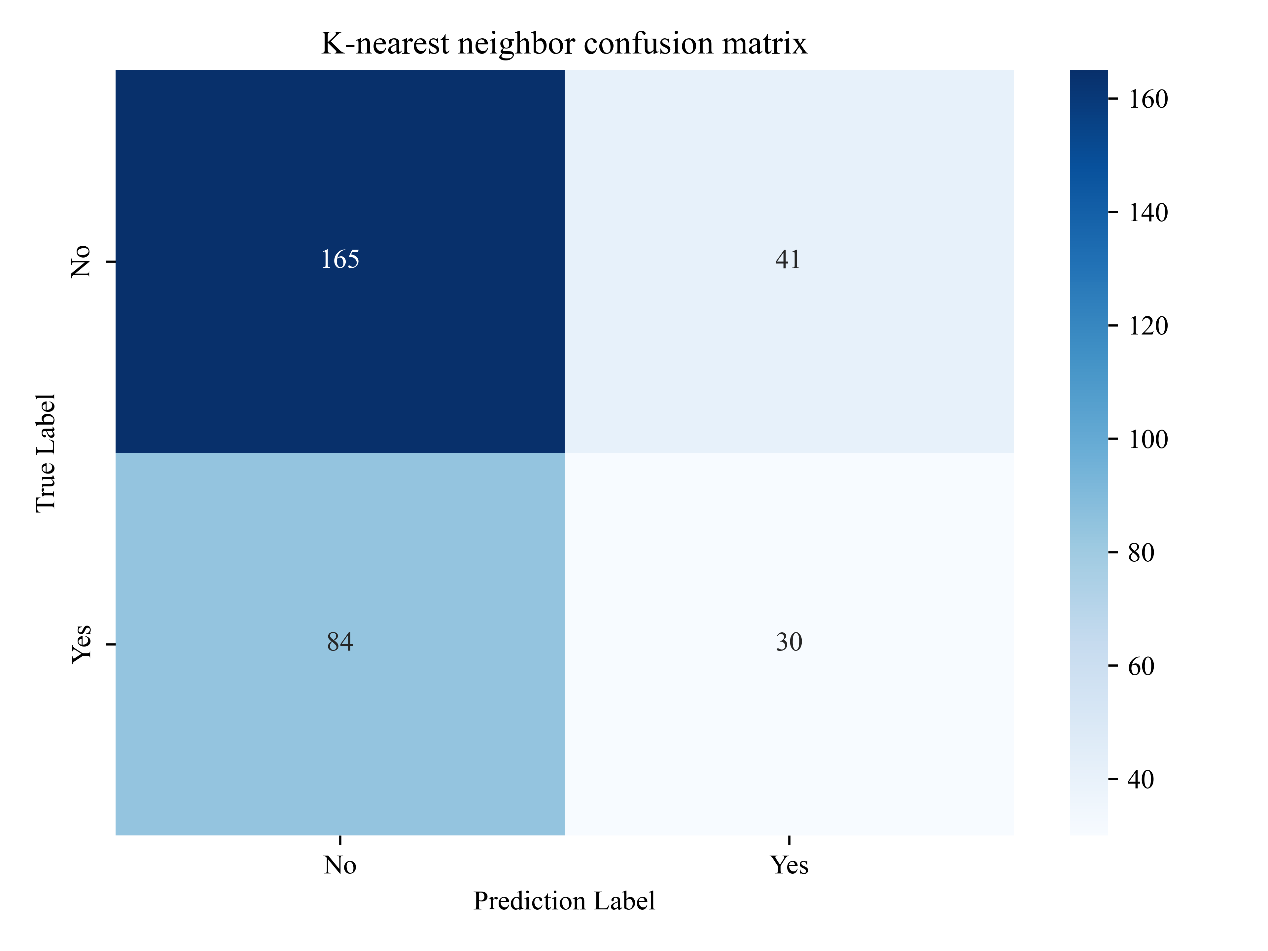


**Figure 5.K-nearest neighbor confusion matrix**


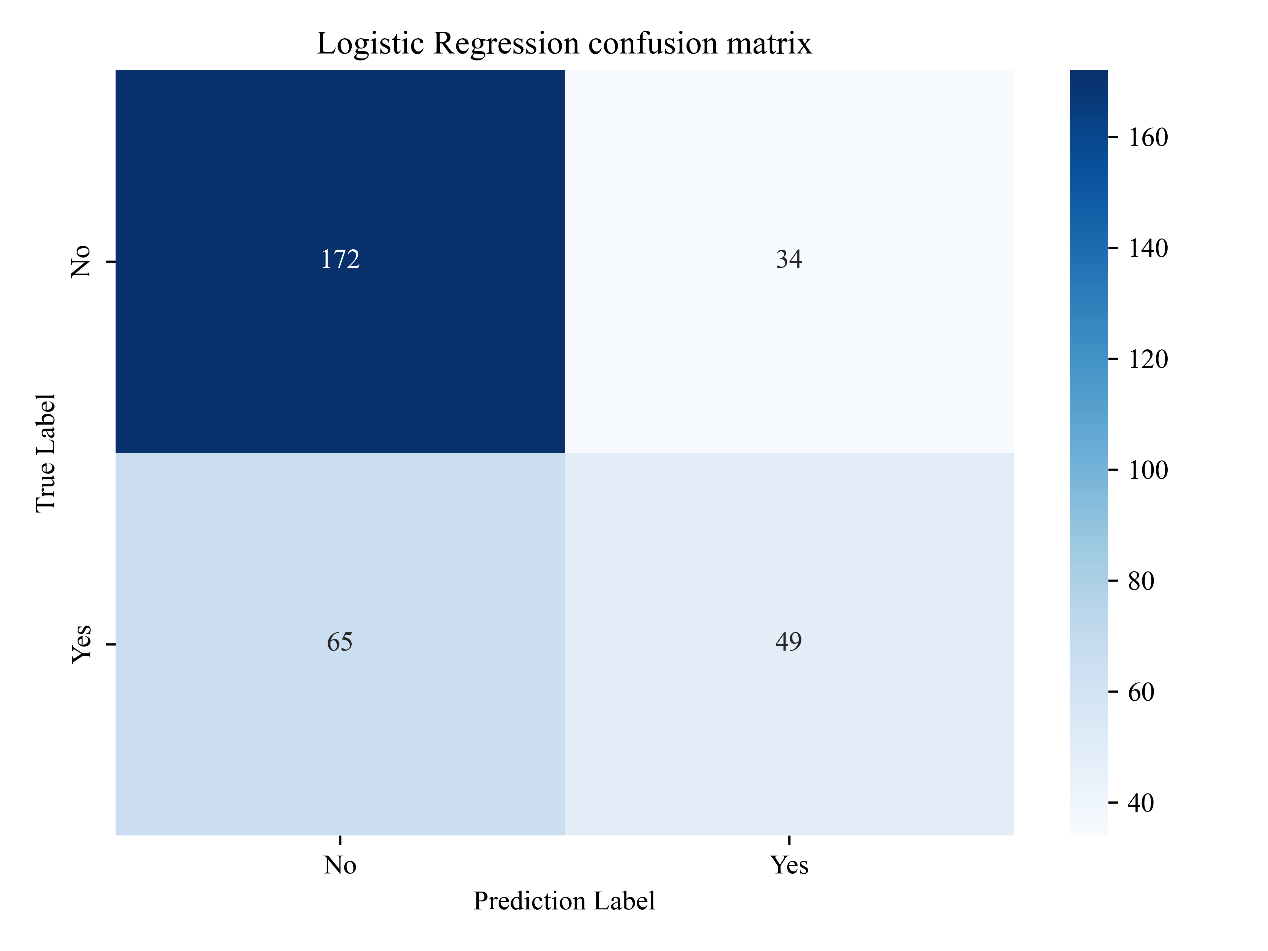


**Figure 6. Logistic Regression confusion matrix**


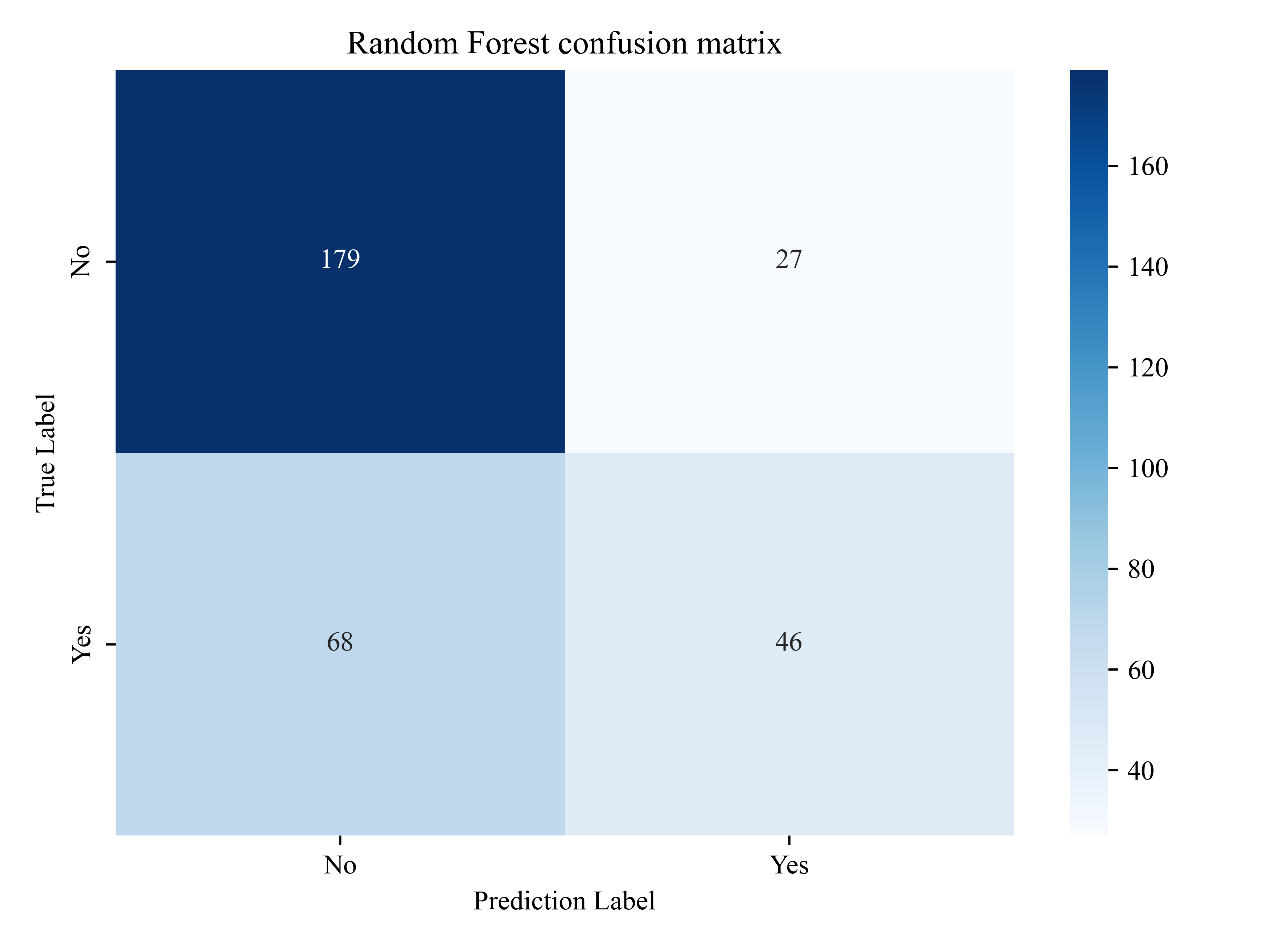


**Figure 7. Random Forest confusion matrix**


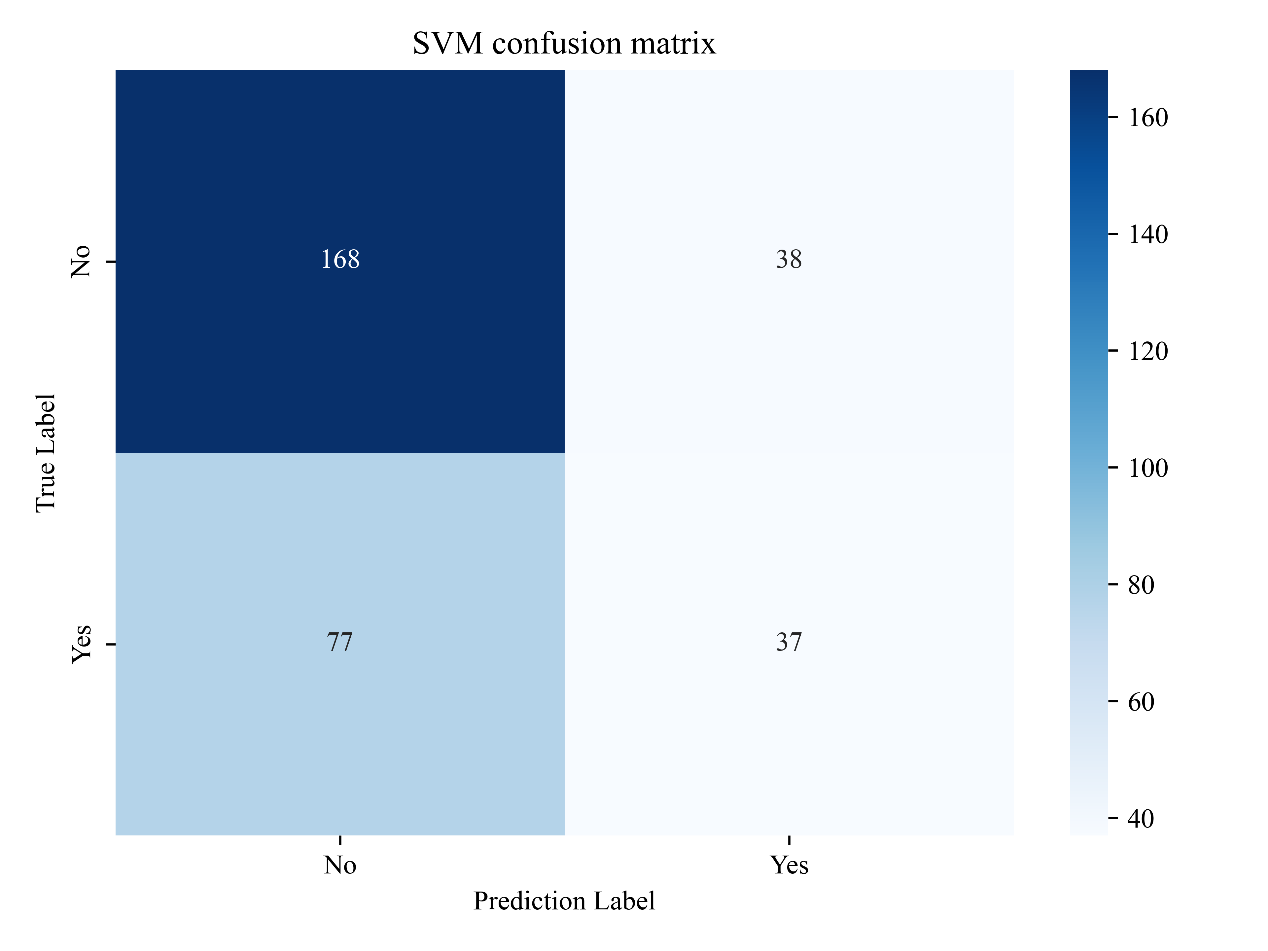


**Figure 8. SVM confusion matrix**


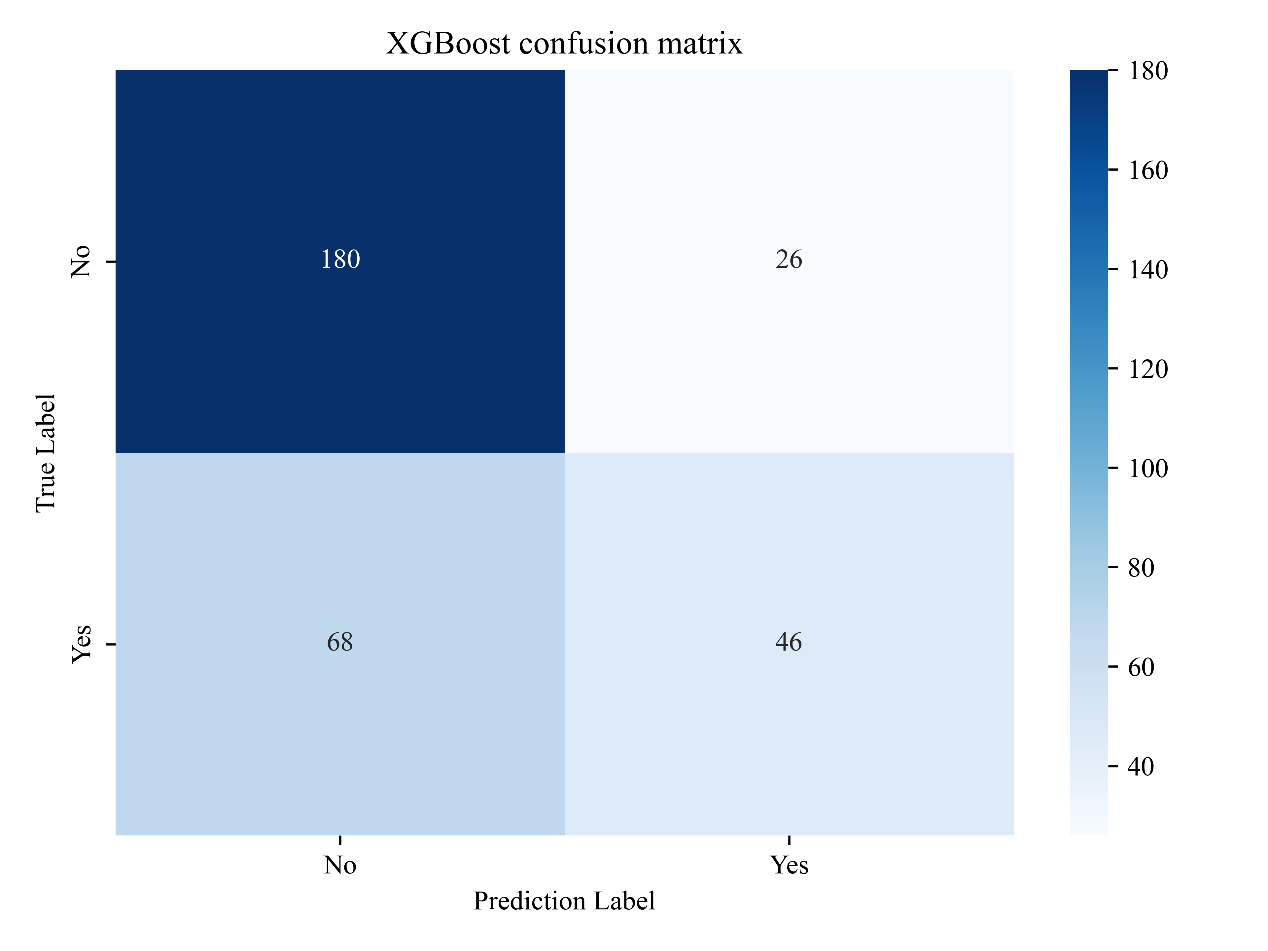


**Figure 9. XGBoost confusion matrix**
